# An easily attachable measurement system of joystick angle in a power wheelchair using IMUs for maneuvering logger

**DOI:** 10.1038/s41598-024-58722-3

**Published:** 2024-04-12

**Authors:** Yi Liu, Jun Suzurikawa

**Affiliations:** 1 College of Mechanical and Electrical Engineering, Harbin Engineering University, Harbin, 150001 China; 2 Department of Assistive Technology, Research Institute, National Rehabilitation Center for Persons With Disabilities, Tokorozawa, Saitama 3598555 Japan

**Keywords:** Health care, Engineering, Disability

## Abstract

Monitoring joystick operations in power wheelchairs (PWCs) is promising for investigating user-wheelchair interaction and providing quantitative measures to assess the user’s driving performance. In this paper, an add-on measurement system, Power Wheelchair Maneuvering Logger (PWhML), is developed to provide an easy-to-implement and cost-effective solution for monitoring the user’s joystick operations in PWCs. The proposed system uses two compact inertial measurement units (IMUs), which are respectively attached to the joystick and wheelchair armrest for movement sensing. A coordinate transformation-based method is proposed to estimate the joystick operating angles using the acceleration data measured by the attached IMUs. The accuracy of the proposed method was thoroughly evaluated under different conditions. The evaluation trials in a stationary PWC reported a mean absolute error (MAE) of 0.59° in the forward/backward direction and 0.64° in the leftward/rightward direction, validating the established geometry model for coordinate transformation. The subsequent driving experiments on outdoor test courses demonstrated the effectiveness and robustness of the proposed method in various terrain conditions (MAE of less than 3°). A clustering analysis based on the t-distributed stochastic neighborhood embedding method correctly categorized different driving activities using the joystick operating angles measured by PWhML. These results indicate that integrating the developed PWhML into PWCs can facilitate a quantitative measurement of the user’s driving behavior, providing valuable insights to identify careless operation patterns and help PWC users to improve driving performance.

## Introduction

Mobility impairments often come with environmental barriers that restrict an individual’s range of daily activities and significantly affect the quality of life^1^. As an alternative to ambulation, wheelchairs provide substantial benefits to individuals with mobility impairments, allowing them to access a broader range of activities, participate in local communities, and gain greater independence^2^. According to an investigation by the Wheelchair Foundation, approximately 131.8 million people with physical disabilities need wheelchairs for mobility assistance^3^. Furthermore, the population of wheelchair users has been increasing by 5.9% per year because of the rapid growth of the global aging population and the fast spread of chronic disorders^4^. In the United States, there are currently over 3.3 million wheelchair users, among whom 1.825 million are aged 65 and older^5^. In recent years, power wheelchairs (PWCs), which use batteries and electric motors for propulsion, have been gaining popularity in helping disabled or elderly individuals with severe motor dysfunctions move around effortlessly and efficiently^6^.

With the increasing prevalence of PWCs, there is a growing concern regarding safety issues^7^. Like using other motorized vehicles, driving PWCs necessitates acquiring operation skills and capability. Although some driving assistance systems^8,9^ and intelligent wheelchair control^10^ were tentatively incorporated into the PWC to facilitate the driving operation in terrain-specific environments, numerous PWC users claimed they had experienced some difficulty driving outside the home (e.g., when facing uneven surfaces such as an inclination) and maneuvering within and through small spaces such as doorways and elevators^11^.

Performance-based assessment methods (e.g., Wheelchair Skills Test) have been developed to evaluate the individual's capability and skills when driving a PWC^12^. In these tests, the participants are instructed to complete standardized driving tasks in controlled or real-world environments. A certified rater evaluates each individual’s driving performance to grade their skill level^13^. The assessment process is a qualitative measurement primarily based on visual observations of a human rater. These tests, therefore, may overlook relevant characteristics of the driving operations and wheelchair dynamics that are essential to the characterization of the user’s driving performance^14^.

An alternative and more cost-effective solution is to use embedded sensors to obtain quantitative data during PWC driving^15^. Some sensor-based measurement systems have been emerging to implement a quantitative evaluation of the user’s performance in wheelchairs^16,17^. Copper et al. installed a customized datalogger in a PWC to measure its speed, travel distance, and duration of use in an unrestricted community environment to determine the activity levels of PWC users^18^. Sonenblum et al. developed a Wheelchair Activity Monitoring Instrument to record the usage information of the PWC and comprehensively compared the difference in the activities between indoor and outdoor environments^19^. Despite the progress in monitoring wheelchair usage information and exploration of wheelchair-environment interaction, the user-wheelchair interaction in PWCs was not explored in these studies.

It is reported that in PWC-related accidents, 33% were directly relate to the user’s careless driving behaviors and insufficient operational skills^20^. Therefore, monitoring joystick operations in PWCs is promising for investigating user-wheelchair interaction and providing quantitative measures to assess the user's driving performance^21^. Suzurikawa et al. quantified the operational burden based on joystick input signals, thereby evaluating the effectiveness of a downhill-turning prevention control for improving PWC safety^22^. Sorrento et al. also explored the possibility of using joystick signals to discriminate the operation characteristics of novice and experienced PWC users^23^. Nevertheless, the signal acquisition depended on refitted joystick controllers requiring additional modification of internal electrical circuits. The research-specific design makes it difficult to apply the measurement methods to other standard PWCs. Rabreau et al. implemented an easy-to-plug solution that integrated IMUs into the PWC to monitor joystick operations, ensuring versatility across a broader range of commercially available PWCs^24^. Despite these advances in design, the joystick operating angles were estimated based on a simple mathematical model that directly subtracts the two accelerometer components. Although this solution was effective for simple maneuvering tasks on flat terrain, more complex joystick operations on uneven/inclined terrain were prone to cause significant estimation errors.

In this paper, we develop an add-on measurement system, Power Wheelchair Maneuvering Logger (PWhML), that uses the precise coordinate transformation of inertial measurement unit (IMU) signals to estimate the joystick operating angles in complex terrains. The proposed method was tested in a series of driving experiments with various terrain conditions, with results demonstrating its accuracy and robustness against terrain inclination. In addition, PWhML can be effortlessly integrated into existing joystick-driven PWC without the need for modifications, providing a low-cost and easy-to-implement solution to monitor the user’s driving behaviors using their own PWC. With the use of the maneuvering logger provided by the PWhML, a statistical clustering method, t-distributed stochastic neighborhood embedding (t-SNE), is introduced to identify the characteristics of joystick operations in different driving activities.

## Methods

### Estimation of joystick operating angles using coordinate transformation matrices

A joystick functions as a two-degree-of-freedom interface in standard PWCs for proportional drive control, which maps the direction and angular displacement of the joystick to the moving direction and speed of a PWC, i.e., the wheelchair is actuated in the direction to which the user points the joystick. The greater the joystick’s angular displacement, the faster the PWC will move.

**Figure 1:**
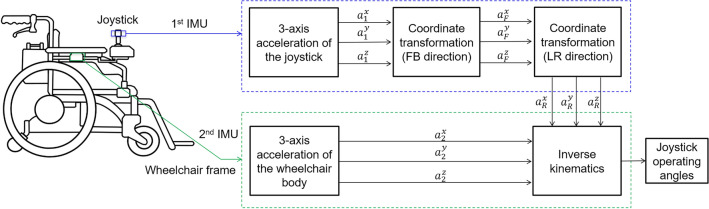
Procedures of the proposed coordinate transformation-based method for estimation of the joystick angles.

The joystick movement can be decomposed into the angular displacements in the directions of forward/backward (FB) and leftward/rightward (LR). For quantification of the user’s operations, a coordinate transformation-based method using the acceleration data of IMUs is proposed to estimate the joystick operating angles in FB and LR directions. The schematic of the proposed method is presented in Fig. 1. Two IMUs are attached to the joystick and wheelchair armrest to measure the acceleration data of the joystick and wheelchair body. The three-axis acceleration data measured on the joystick are sequentially transformed using the coordinate transformation in FB and LR directions. An additional IMU attached to the wheelchair armrest measures the three-axis acceleration of the wheelchair body to compensate for the wheelchair acceleration and inclination on uneven terrains. By combining the transformed acceleration on the joystick and the wheelchair body, the joystick operating angles in the FB and LR directions can be deduced by solving an inverse kinematics problem.

**Figure 2:**
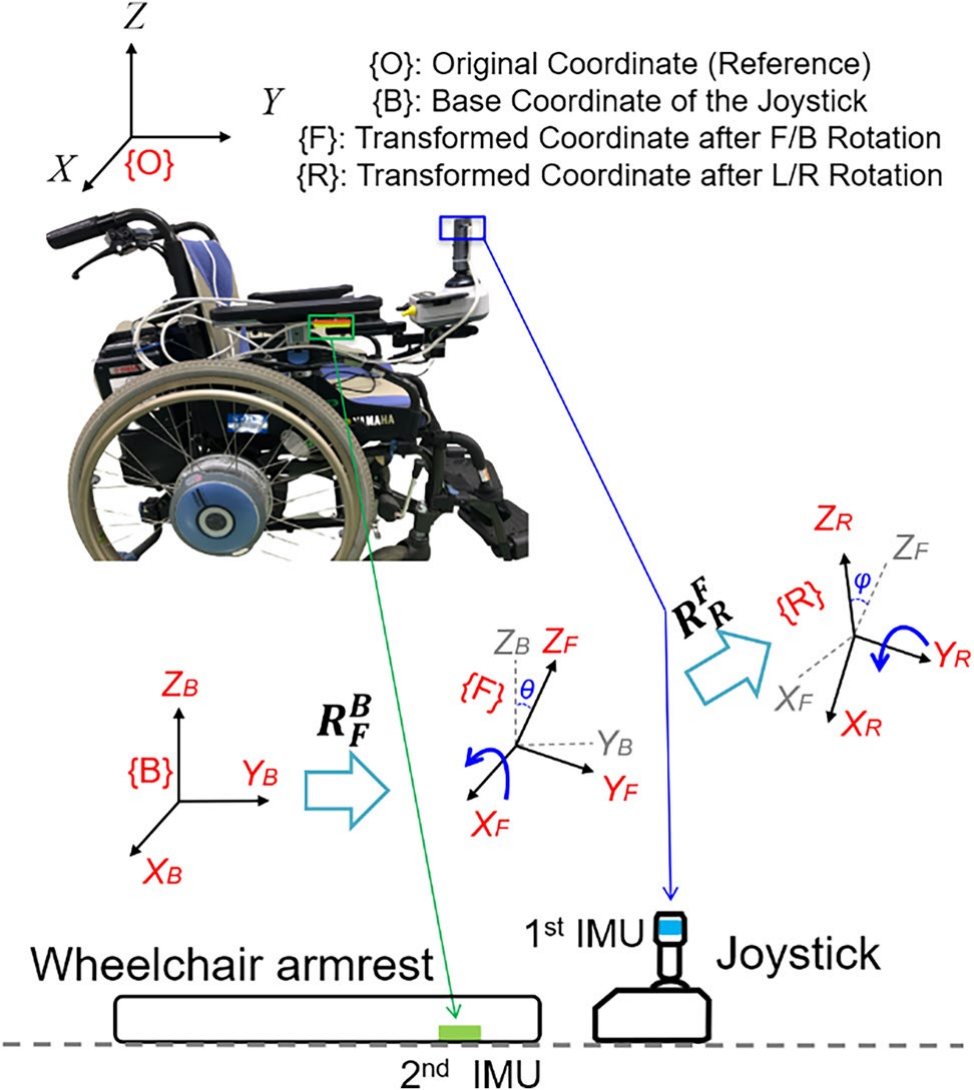
Transformation geometry for estimation of the joystick operating angles.

Figure 2 illustrates the transformation geometry of the joystick spatial coordinates, where the reference coordinate is defined as {O}. An IMU is attached to the joystick to measure its three-axis acceleration, the coordinate of which is defined as {B}. In a stationary state, the joystick remains in the resting position at the center, where the orientation of the base coordinate {B} is consistent with the reference coordinate {O}. After the joystick is pushed away, the 3D spatial coordinate of the embedded IMU is rotated, and the three-axis acceleration measured by the IMU varies with gravity. The joystick operation can be decomposed into the sequential rotations in FB and LR directions around the resting position, the rotation matrices of which are described as:

1 $$ R_{F}^{B} = \left[ {\begin{array}{*{20}c} 1 & 0 & 0 \\ 0 & {\cos \left( { - \theta } \right)} & { - \sin \left( { - \theta } \right)} \\ 0 & {\sin ( - \theta )} & {\cos ( - \theta )} \\ \end{array} } \right] $$

2 $$ R_{R}^{F} = \left[ {\begin{array}{*{20}c} {\cos \varphi } & 0 & { - \sin \varphi } \\ 0 & 1 & 0 \\ {\sin \varphi } & 0 & {\cos \varphi } \\ \end{array} } \right] $$

where θ and φ are the joystick operating angles in the FB and LR directions. With reference to base coordinate {B}, the rotated coordinate {R} is obtained by rotating − θ around the X axis in the FB direction and φ around the Y axis in the LR direction.

When the PWC remains still on a flat surface, only the gravitational acceleration is applied to the wheelchair. The transformation of the three-axis acceleration from the base coordinate {B} to the transformed coordinate {R} is expressed by:

3 $$ \left[ {\begin{array}{*{20}c} 0 \\ 0 \\ { - g} \\ \end{array} } \right] = R_{F}^{B} \cdot R_{R}^{F} \cdot \left[ {\begin{array}{*{20}c} {a_{1}^{x} } \\ {a_{1}^{y} } \\ {a_{1}^{z} } \\ \end{array} } \right] $$

where ${[{a}_{1}^{x} {a}_{1}^{y} {a}_{1}^{z}]}^{T}$ is the three-axis acceleration measured by the IMU attached to the joystick, and g is the gravitational acceleration equal to 9.8 m/s^2^.

However, the acceleration vector in the base coordinate {B} may not always be identical to [0, 0, − g]^T^ because of terrain inclination and wheelchair movements. An additional IMU is attached to the armrest of the wheelchair to measure the dynamics of the wheelchair and compensate for these disturbances. Therefore, Eq. (3) can be rewritten as:

4 $$ \left[ {\begin{array}{*{20}c} {a_{2}^{x} } \\ {a_{2}^{y} } \\ {a_{2}^{z} } \\ \end{array} } \right] = R_{F}^{B} \cdot R_{R}^{F} \cdot \left[ {\begin{array}{*{20}c} {a_{1}^{x} } \\ {a_{1}^{y} } \\ {a_{1}^{z} } \\ \end{array} } \right] $$

Because $R_{F}^{B} \cdot \left( {R_{F}^{B} } \right)^{T} = E$, we can get $\left( {R_{F}^{B} } \right)^{T} = \left( {R_{F}^{B} } \right)^{ - 1}$. Likese, $\left( {R_{R}^{F} } \right)^{T} = \left( {R_{R}^{F} } \right)^{ - 1}$. Therefore, Eq. 4 is equal to:

5 $$ \left( {R_{R}^{F} } \right)^{T} \cdot \left( {R_{F}^{B} } \right)^{T} \cdot \left[ {\begin{array}{*{20}c} {a_{2}^{x} } \\ {a_{2}^{y} } \\ {a_{2}^{z} } \\ \end{array} } \right] = \left[ {\begin{array}{*{20}c} {a_{1}^{x} } \\ {a_{1}^{y} } \\ {a_{1}^{z} } \\ \end{array} } \right]. $$

where $$\left( {R_{R}^{F} } \right)^{T} \cdot \left( {R_{F}^{B} } \right)^{T} =$$

6 $$ \left[ {\begin{array}{*{20}c} {\cos \varphi } & { - \sin \varphi sin\left( { - \theta } \right)} & { - \sin \varphi cos\left( { - \theta } \right)} \\ 0 & {cos\left( { - \theta } \right)} & {sin\left( { - \theta } \right)} \\ { - \sin \varphi } & { - \cos \varphi sin\left( { - \theta } \right)} & {\cos \varphi cos\left( { - \theta } \right)} \\ \end{array} } \right] $$

By substituting the acceleration vectors obtained from the two IMUs to solve the inverse kinematics problem, the joystick operating angles θ and φ are obtained, as denoted by:

7 $$ \theta = \arctan \left( {\frac{{a_{2}^{y} }}{{a_{2}^{z} }}} \right) - arcsin\left( {\frac{{a_{1}^{y} }}{{\sqrt {\left( {a_{2}^{y} } \right)^{2} + \left( {a_{2}^{z} } \right)^{2} } }}} \right) $$

8 $$ \varphi = \arcsin \left( {\frac{{a_{1}^{x} }}{{\sqrt {\left( {\sin \left( { - \theta } \right) \cdot \left( { - a_{2}^{y} } \right) + {\text{cos}}\left( { - \theta } \right) \cdot a_{2}^{z} } \right)^{2} + \left( {a_{2}^{x} } \right)^{2} } }}} \right) - \arctan \left( {\frac{{a_{2}^{x} }}{K}} \right) $$

### Configuration of PWhML

**Figure 3:**
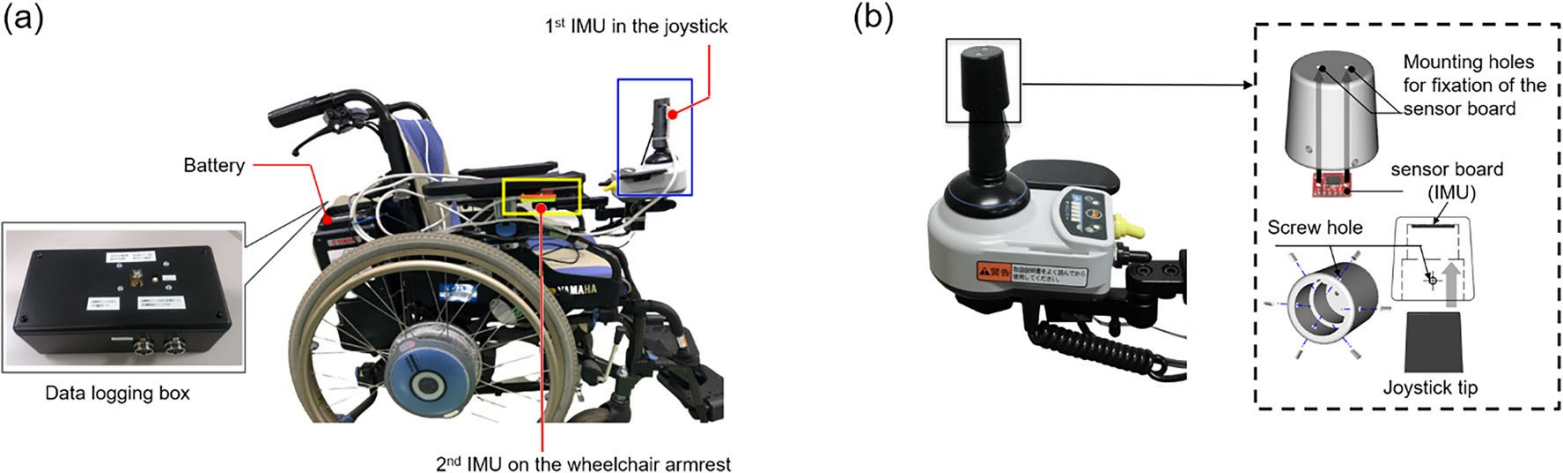
Overview of PWhML mounted on a test PWC. (**a**) PWhML Set-up. (**b**) Affixation of the IMU on the joystick.

PWhML is designed to be an add-on unit that can be installed on any commercially available PWC with a standard joystick controller. For proof of concept, it is installed on a standard joystick-driven PWC (JWX-1 PLUS+ , Yamaha Motor Co., Ltd., Japan) in this study. As depicted in Fig. 3a, PWhML uses two 6-axis IMUs (GY-LSM6DS3) to measure the acceleration of the joystick and wheelchair. A portable data logging box, which contains a microcontroller (Arduino MKR ZERO, Arduino.cc, Italy) and the peripheral electronics, is placed in the back pocket of the PWC.

The data logging box can simultaneously receive the joystick voltage outputs through a modified electrical circuit of the PWC control board. These signals are converted to the angular displacements of the joystick in the FB and LR directions, which are used to evaluate the accuracy of the proposed coordinate transformation-based method. All data are simultaneously sent to the microcontroller via serial communication at a sampling rate of 30 Hz and automatically saved in the data logging box. Each data sample is composed of eight components: six acceleration data of two IMU sensors (${a}_{1}^{x}$, ${a}_{1}^{y}$*, *${a}_{1}^{z}$*, *${a}_{2}^{x}$*,* ${a}_{2}^{y}$, and ${a}_{2}^{z}$) and two joystick voltage outputs (V_fb_ and V_lr_).

A hand-operated joystick is placed on the right side of the wheelchair armrest to enable the user to control the PWC movements. An IMU is fixed to the joystick tip using a 3D-printed cover cap to measure joystick movements, as depicted in Fig. 3b. This attachable cap facilitates an easy mount on any other joystick-driven PWCs having different sizes and configurations, providing a cost-effective and convenient solution for monitoring joystick operations of PWCs.

### Test courses and experimental set-up for accuracy evaluation

**Figure 4:**
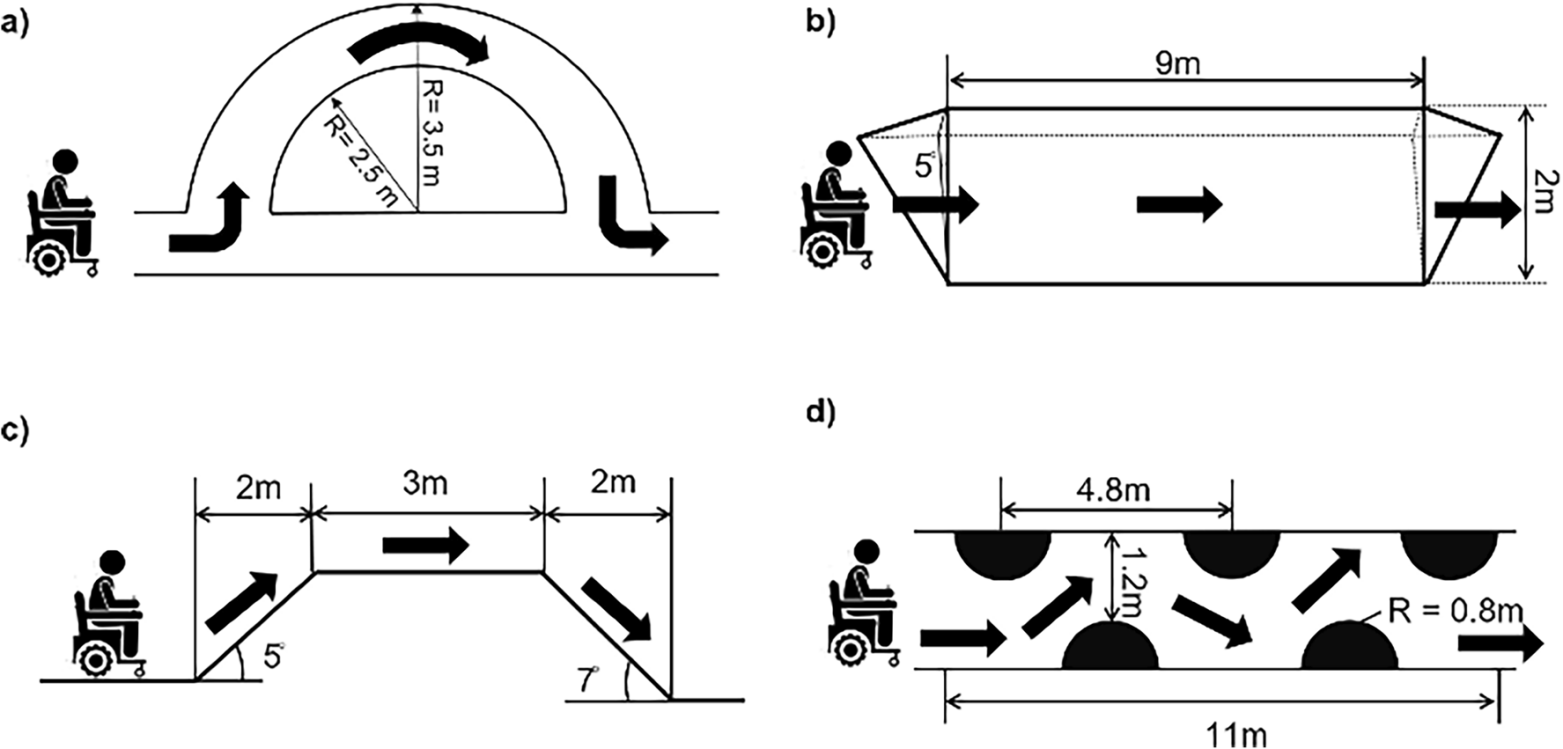
Single-task test course. (**a**) Curving (**b**) Side slope with 5° inclination (**c**) Up/down slope (**d**) Slalom driving.

Four distinct driving tasks requiring different joystick maneuvering are designed to demonstrate the effectiveness of the proposed method on various terrains, as depicted in Fig. 4: (a) curve with a radius of 2.5 m, (b) roll 9 m across 5° side slope, (c) ascend 5° incline and then descend 7° incline, and (d) continuous slalom driving across five fixed obstacles. These tasks are primarily selected from the Wheelchair Skills Test^25^. However, minor modifications are made to increase the maneuvering load and operational difficulty to rigorously evaluate the accuracy of the proposed method even in more challenging scenarios.

**Figure 5:**
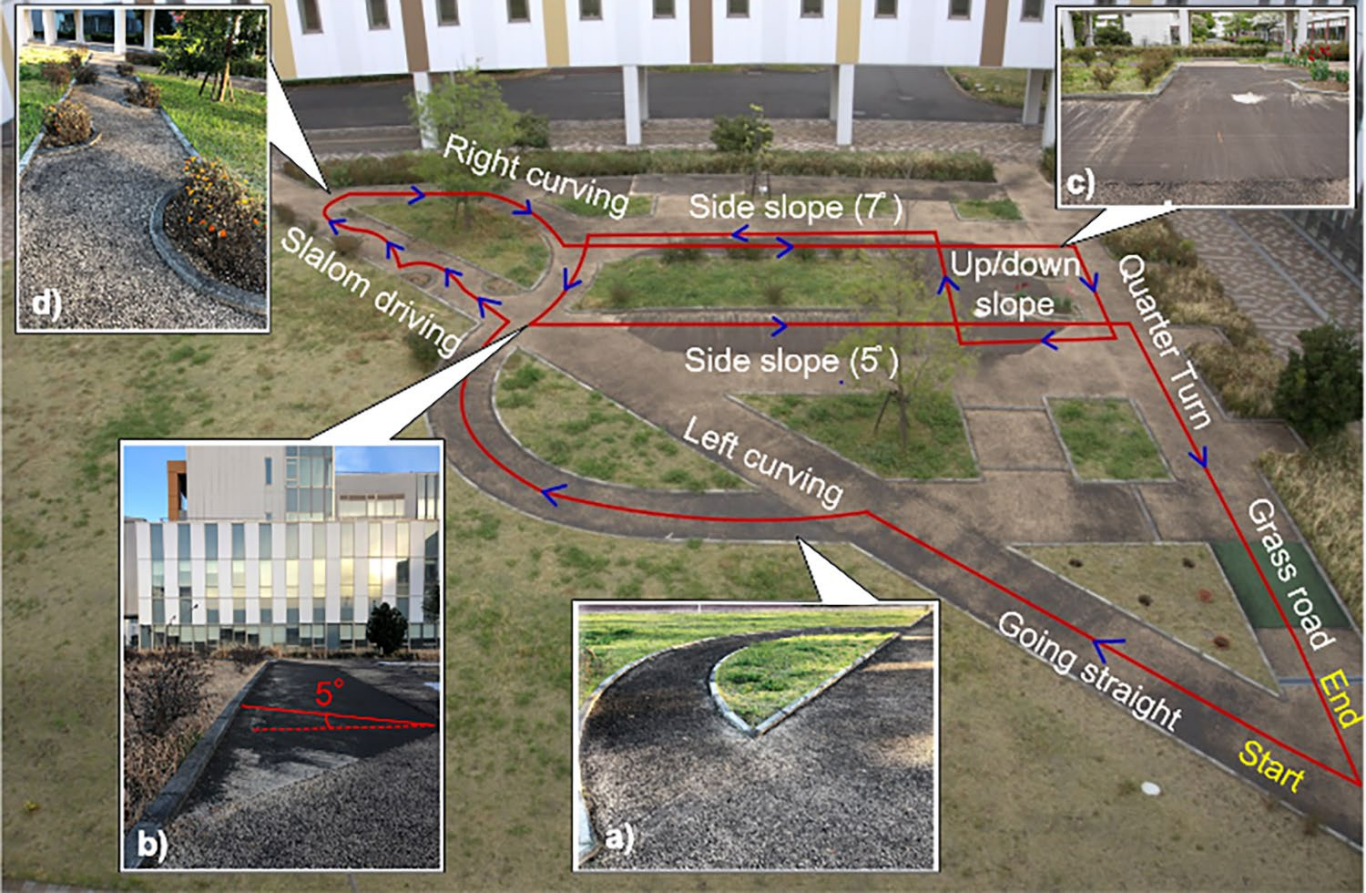
Multiple-task driving course on the outdoor wheelchair training ground.

A multiple-task driving course performed on an outdoor wheelchair training ground is also designed to evaluate the effectiveness of the proposed method for continuous measurement of joystick operations. As depicted in Fig. 5, it includes the most common activities in the daily driving of PWCs, and various types of wheelchair maneuvering are assigned to be conducted by the participants on uneven terrains.

This study was conducted according to the guidelines of the Declaration of Helsinki and approved by the Institutional Review Board of National Rehabilitation Center for Persons with Disabilities, Japan (#2021-130). In this study, four healthy participants (three males and one female, average age: 30.8 ± 15.5 years old, average height: 167.5 ± 5.9 cm, average weight: 63.5 ± 6.5 kg) were enrolled in the experiment after providing a written informed consent. The demographic information of the participants in the experiments is shown in Table 1. The participants were instructed to perform the assigned four driving tasks and multiple-task driving successively, and the same experimental procedure was repeated five times. During the process, the acceleration data measured by the 2 IMUs were collected using PWhML. The raw acceleration data were processed using a 25th-order median filter^26^ to remove the white Gaussian noise internally generated by the IMUs and spike noises caused by a large impact while moving^27^. The filtered three-axis acceleration data were transformed into the time-series joystick angles in FB and LR directions using the proposed coordinate transformation-based method.

**Table 1:** Demographic information of the participants in the experiments.

Participant	Gender	Age (years)	Height (cm)	Weight (kg)
#1	Female	54	164	54.5
#2	Male	23	163	63.0
#3	Male	23	167	67.2
#4	Male	23	176	69.1

### Driving activity clustering

**Figure 6:**
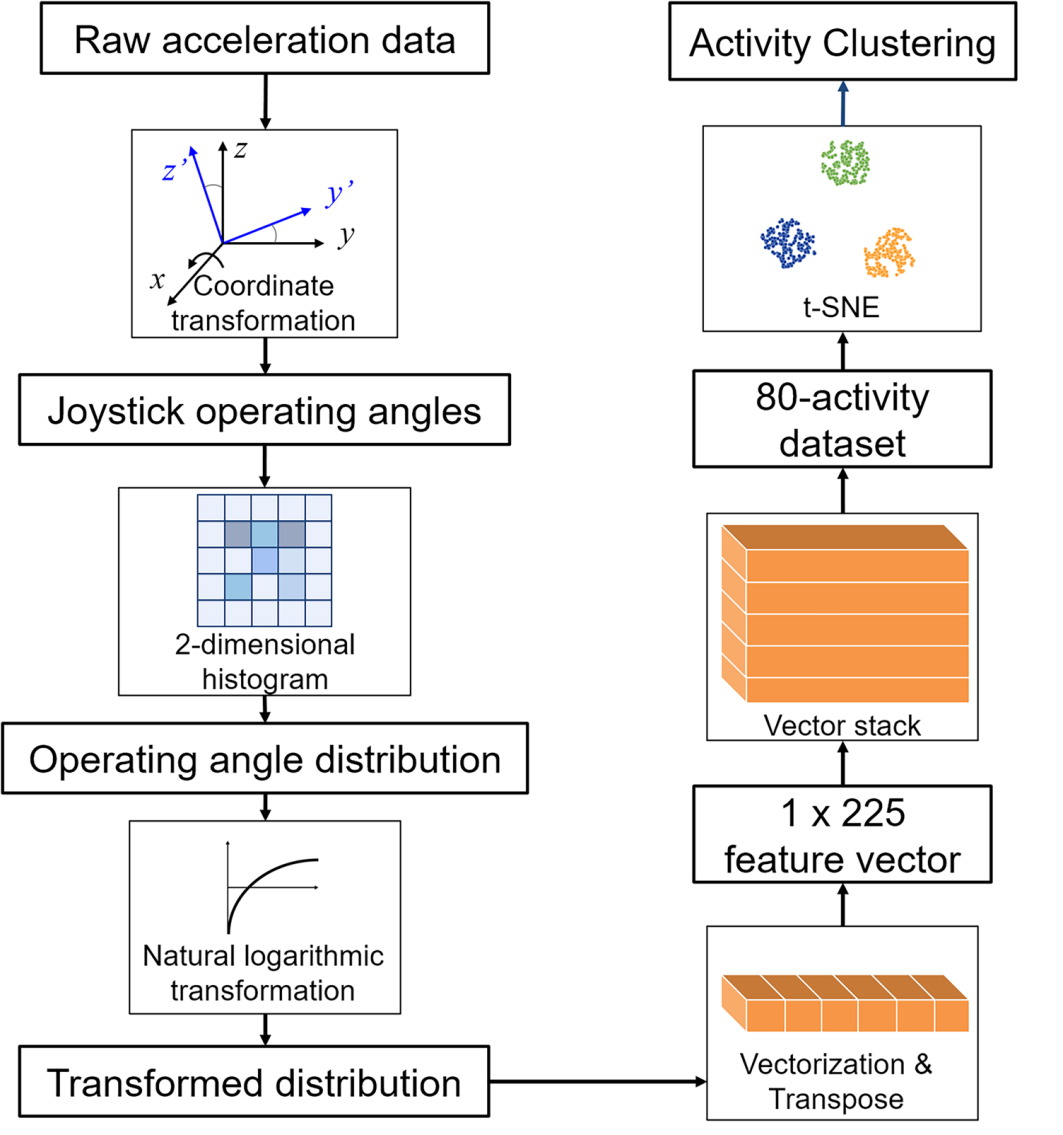
Processing procedures for activity clustering.

Finally, with the acceleration measured on the joystick and wheelchair body in each driving activity using the IMUs, we characterize the user’s operation patterns for different driving tasks. Figure 6 illustrates the processing procedures for activity clustering using the recorded operation log. The time-series operating angles are subsequently transformed into a two-dimensional histogram with a bin width of 2° to describe the distribution of operating angles in the test driving. Each value of the 2D histogram represents the temporal amount of the operating status corresponding with the FB and LR angles of the bin. As the operation angles mainly fall in the range of (− 10°, 20°) in the FB direction and (− 15°, 15°) in the LR direction, the distribution map is segmented by 15 × 15 bins. After transformation, vectorization and transpose are implemented to flatten the distribution map (15 × 15 bins) into a row vector (1 × 225), regarded as the driving activity’s pattern features. This study creates a dataset containing 80 independent activities (four participants, four assigned driving tasks, and five trials for each task) for clustering. Each activity in the dataset can be regarded as a data point in the high-dimensional space. A t-SNE-based clustering method^28^ is implemented to visualize the clustering of the driving activities in a two-dimensional plane.

## Results

### Characterization of the proposed coordinate transformation-based estimation method

#### Estimation accuracy in a stationary PWC

**Figure 7:**
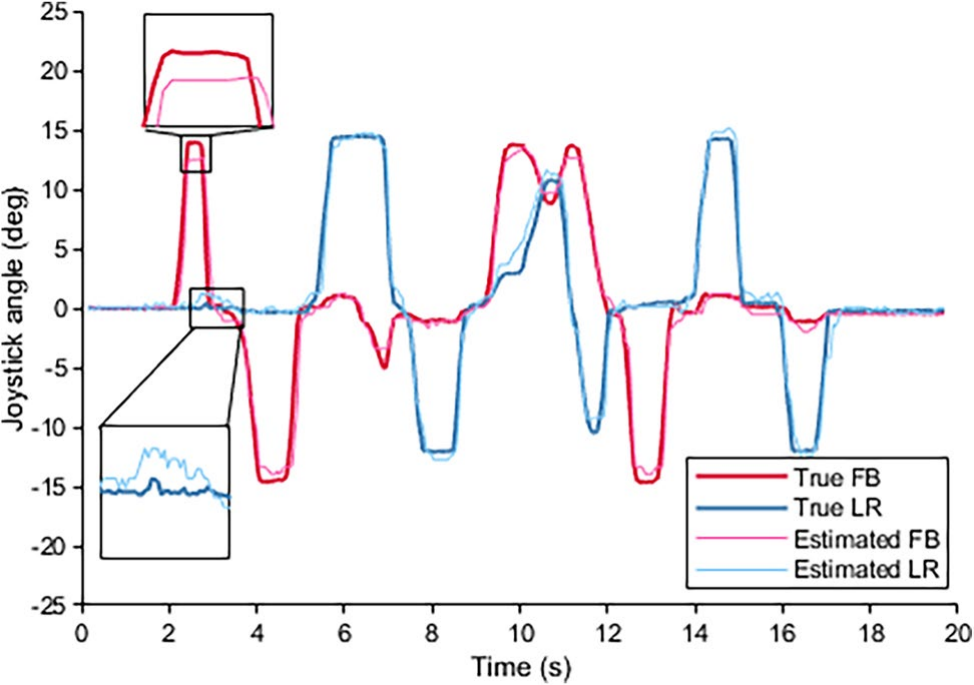
Estimation of the joystick operating angles in a static condition.

The test trial was first conducted on a stationary PWC to validate the geometric model of the proposed coordinate transformation method. During the experiment, the joystick was pushed toward the FB and LR directions successively. The actuation of the PWC was powered off and the autobrake was applied to prevent the wheels from moving. As depicted in Fig. 7, the joystick angles estimated by the proposed method are comparable to the actual values directly obtained from the joystick command signals. The mean absolute errors (MAEs) between the estimated and actual angles in the static condition were 0.59° in the FB direction and 0.64° in the LR direction. The coordinate misalignment of IMUs and backlash in the rotational axes of the joystick interface may have contributed to these minor errors. Due to mechanical looseness, the operating force applied to the joystick caused slight position change of the joystick platform, resulting in a shift of the primary IMU from the calibrated position. As observed in the partial enlarged view of Fig. 7, the peak deviation of the true and estimated joystick operating angles in the FB direction was nearly uniform, indicating that the estimation error might be caused by the misalignment of the base coordinate of IMUs. Additionally, it is noted that when releasing the joystick from FB direction, the estimation error in the LR direction was increased due to backlash in the rotational axes of the joystick interface. Despite these minor errors, the results demonstrated the effectiveness of the proposed coordinate transformation-based method for estimating joystick operating angles and confirmed its accuracy in a static condition.

#### Estimation accuracy in a moving PWC

**Figure 8:**
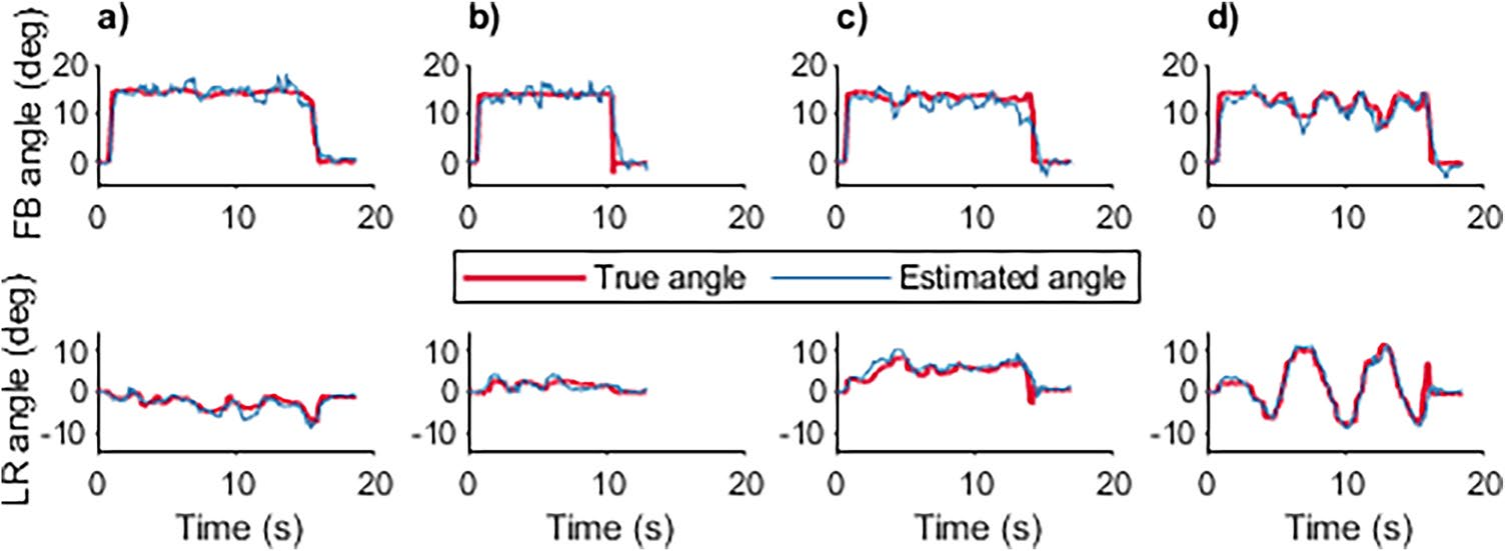
Experimental results for single-task driving. (**a**) Curving (**b**) Side slope with 5° inclination (**c**) Up and down slope (**d**) Slalom driving.

Figure 8 illustrates the actual and estimated joystick operating angles during the test drive in different conditions. The overall estimation has comparable accuracy to that in the static condition, although the motion-induced inertia and uneven terrain partly caused estimation errors. For example, in the task of up/down slope (Fig. 8c), a noticeable angle difference in FB direction was observed around 11 s. As the participant suddenly released the joystick to slow down the PWC when descending the slope, the opposite acceleration trend on the joystick and wheelchair body resulted in a significant lag in estimating the joystick FB operating angle. Nevertheless, these extreme situations account for a limited period throughout the driving process, thereby having little effect on the overall performance of the proposed method.

**Figure 9:**
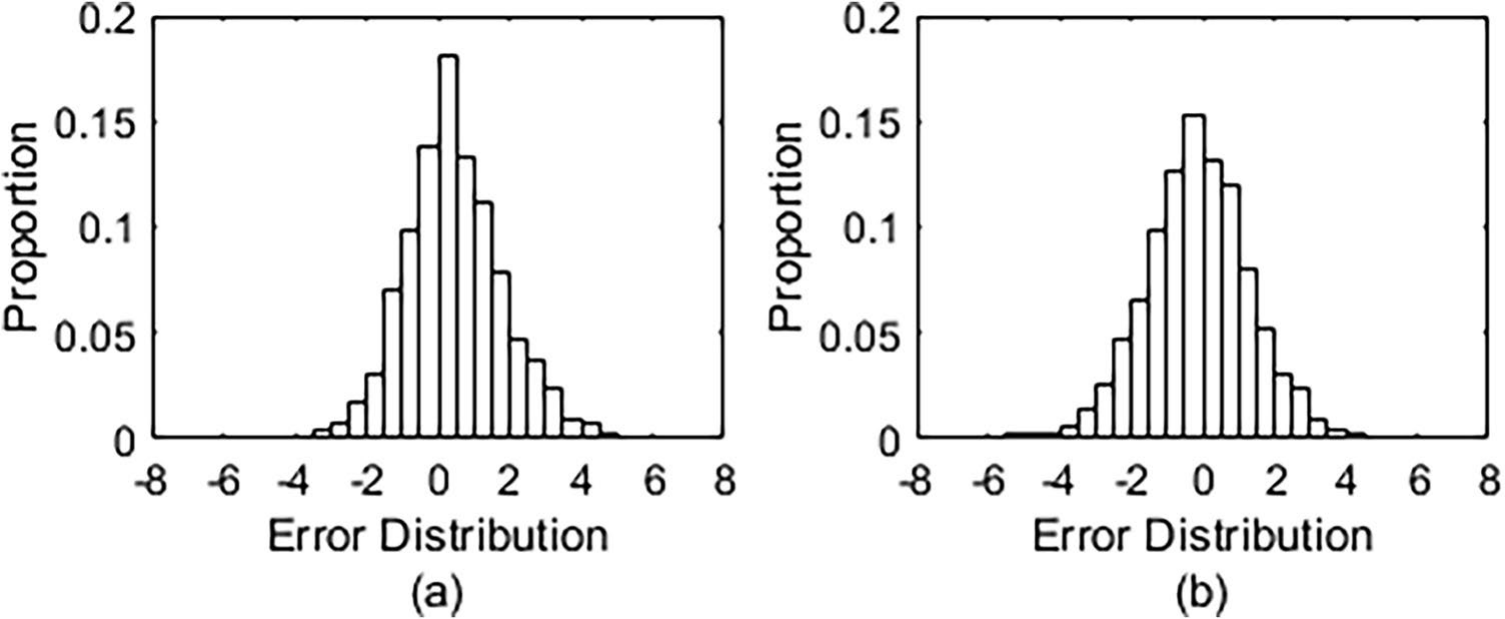
Distribution of the estimation error. (**a**) FB direction. (**b**) LR direction.

The experimental trials were conducted on the outdoor wheelchair training ground to simulate the most common challenging scenarios that appear in daily community driving and further evaluate the performance of the proposed method for continuous measurement. Figure 9 illustrates the distribution of estimation errors in FB and LR directions obtained from one trial of the assigned multiple-task driving. Both of them have a typical Gaussian distribution. The estimation errors in the range of ± 4° are 97.6% in the FB direction and 98.4% in the LR direction. Furthermore, the 90th percentile values for the estimation errors in the FB and LR directions are 2.61° and 2.40°.

**Figure 10:**
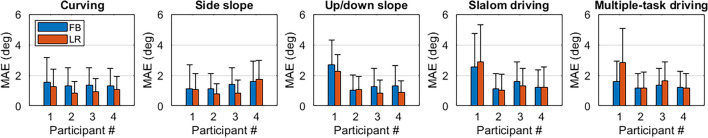
MAEs of the proposed method in different tasks.

The same experimental procedure was repeated five times by each of the four participants to evaluate the overall performance of the proposed method. Figure 10 summarizes the MAEs and standard deviations of the angle estimation errors in each driving task. Across all test courses, the estimation errors of the proposed method fell within a narrow range (less than 3°) in both the FB and LR directions, even on the uneven terrains with an inclination and considerable motion-induced inertia.

### Clustering of driving activities

**Figure 11:**
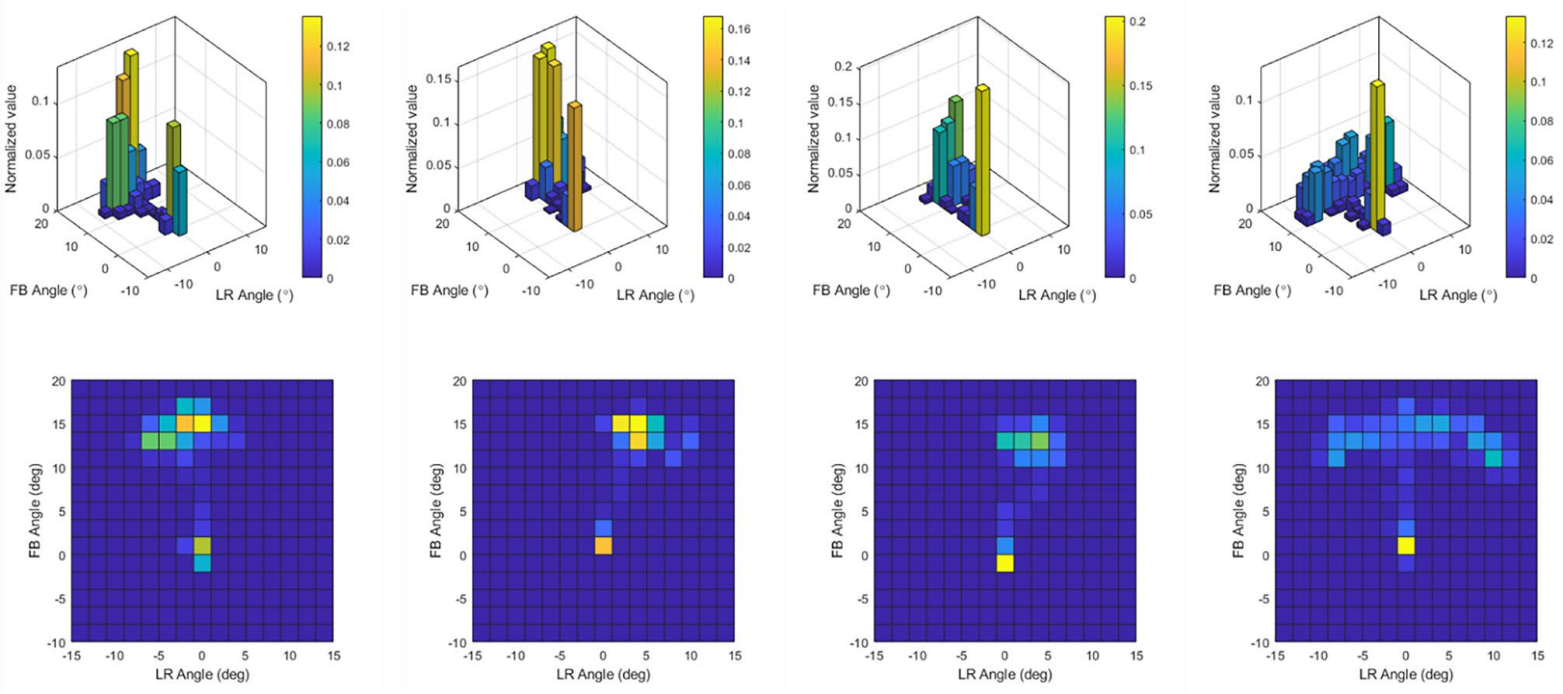
Histogram distribution of the joystick operating angles in different tasks. (**a**) Curving. (**b**) Side slope with an inclination of 5̊. (**c**) Up/down slope. (**d**) Slalom driving.

A comprehensive analysis of joystick operation data enables identification of the characteristics of the operation patterns and discrimination between different driving activities. The time-series operating joystick angles were initially converted to the color-coded distribution map to visualize the characteristics of operation patterns in different driving activities. Figure 11 illustrates an example of the distribution of the joystick operating angles in single-task trials obtained from one participant. The distribution of the operating angles varied among different driving tasks and indicated the possibility of identifying the specific operation pattern in different driving activities.

**Figure 12:**
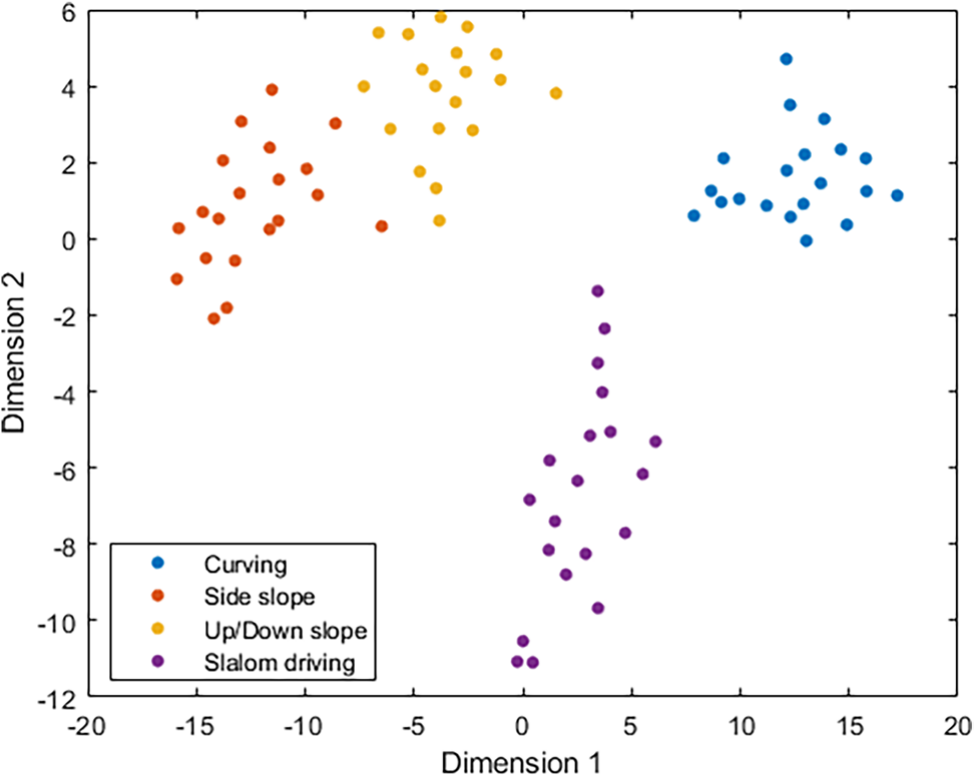
Activity clustering using the t-SNE.

For a demonstration, the operation logs obtained from the single-task experimental trials that contain 80 driving activities in total were used to create a dataset for activity clustering. Following the procedures described in the “Method” Section, the t-SNE algorithm with a learning rate of 50 and perplexity of 15 was implemented to visualize the clustering of the recorded 80 driving activities. As depicted in Fig. 12, the driving activities of the same task are clustered together across different participants, while those of different tasks are well separated from each other.

## Discussion

The measurement and analysis of joystick operation in PWC driving provides possibility to distinguish the variations of the user’s performance among different driving scenarios, further providing valuable insights into potential factors influencing individual performance and driving safety. As indicated in Fig. 10, the estimation accuracy of the proposed method was moderately affected by the user’s maneuvering characteristics. Compared to other three individuals, Participant #1 had a comparable performance in the tasks of curving and side slope that mainly require forward moving with slight adjustment in left/right direction. However, for those tasks requiring advanced joystick maneuvering to control the wheelchair speed and avoid obstacles (up/down slope and slalom driving), the average and standard deviation of the estimation errors of Participant #1 were more significant. The abrupt joystick operation pattern (e.g., sudden release of the joystick to decelerate the wheelchair) may contribute to the reduced estimation accuracy and is detrimental to the driving safety of PWCs. These negative maneuvering factors should be recognized and prevented so as to improve the user’s performance in daily PWC driving.

Despite these significant benefits and promising functions that PWhML provides, some limitations of this study need to be noted and addressed in the near future. Although the effectiveness and robustness of the PWhML were demonstrated under different driving conditions, its accuracy need to be further improved when estimating the joystick operating angles in atypical situations (e.g., the user abruptly released the joystick to decelerate the wheelchair as shown in Fig. 8c). Using a complementary filter to fuse the accelerometer and gyroscope data is a promising treatment to enhance the overall accuracy of the PWhML. In addition, the variations in the performance of different users were statistically analyzed and compared, which provides insights into identifying potential factors related to the individual’s driving performance. Nevertheless, the quantitative criteria for assessing users’ driving performance and skills have not been thoroughly explored in this study. Future research will delve into a more comprehensive analysis of operation logs to establish such criteria for evaluating the user’s driving performance and further validate the effectiveness of the developed PWhML.

## Conclusion

This study developed an add-on power wheelchair maneuvering logger, PWhML, to provide an easy-to-implement and cost-effective solution for quantitatively measuring the user’s joystick operations in PWCs. The configuration of PWhML can be completed by simply attaching two IMUs to the joystick and wheelchair body. Because the process does not require any modifications of the wheelchair structure and internal electrical circuits, PWhML can be effortlessly integrated into any other joystick-driven PWCs. These characteristics (i.e., easy-to-install, low-cost, and compatible with other joystick-driven PWCs) greatly facilitate its practical implementation in real-life environments.

A coordinate transformation-based method was proposed to accurately estimate the joystick operating angles using the acceleration data of the attached IMUs. The accuracy of the proposed method was thoroughly evaluated under different driving conditions. The evaluation test on a stationary PWC validated the established geometry model for coordinate transformation. Subsequently, the driving experiments on the outdoor wheelchair training ground demonstrated its effectiveness and robustness for measurement on various terrains. Moreover, integrating PWhML into PWCs also permits characterization of the user’s operation patterns. An operation pattern analysis using t-SNE clustering method correctly categorized different driving activities from the recorded maneuvering logs, demonstrating the feasibility of identifying joystick operating patterns and monitoring user’s driving status using PWhML. With these promising characteristics as a skills testing and measuring tool, PWhML has the potential to be an alternative to the existing measures that rely primarily on human raters’ observations for monitoring individual’s PWC driving.

## Data Availability

The data that support the findings of this study are available upon reasonable request to the corresponding author.
